# Retroperitoneal Metastatic Apocrine Gland Ductal Adenocarcinoma in a Beagle Dog

**DOI:** 10.3390/vetsci9050234

**Published:** 2022-05-12

**Authors:** Chang-Hwan Moon, Hyun-Ah Min, Hae-Beom Lee, Seong-Mok Jeong, Dae-Hyun Kim

**Affiliations:** Department of Veterinary Surgery, College of Veterinary Medicine, Chungnam National University, 99 Daehak-ro, Yuseong-gu, DaeJeon 34134, Korea; moonch0208@gmail.com (C.-H.M.); minhyeonah@naver.com (H.-A.M.); seatiger76@cnu.ac.kr (H.-B.L.)

**Keywords:** apocrine gland ductal adenocarcinoma, distant metastasis, dogs, retroperitoneal tumor, immunohistochemistry

## Abstract

Tumors of sweat glands usually originate from apocrine glands and can develop throughout the body but are rare in dogs. This report describes the retroperitoneal metastasis of primary cutaneous apocrine adenocarcinoma. An 8-year-old, spayed female beagle dog, weighing 11.7 kg, presented with a history of anorexia, hypodynamia, and weight loss. Clinical examination, radiography, ultrasonography, and computed tomography revealed a skin mass on the dorsum of the right metatarsal region, an enlarged ipsilateral popliteal lymph node, and a retroperitoneal mass. Fine-needle aspiration cytology of the popliteal lymph node suggested metastasis of an apocrine sweat gland tumor. Surgical excision of the skin mass, popliteal lymph node, and retroperitoneal mass was performed. The retroperitoneal mass was diagnosed as a metastasis of primary cutaneous apocrine adenocarcinoma. Immunohistochemistry revealed that the tumor cells were positive for cytokeratin 7 but negative for cytokeratin 20 and S100 proteins. There were no postoperative complications, except for temporary hindlimb edema, including local recurrence or metastasis, in the 6-month postoperative follow-up period. This case illustrates that although malignant apocrine gland tumors are rare in dogs, a wide resection of primary cutaneous apocrine gland adenocarcinomas is recommended because of the risk of local invasion or distant metastasis.

## 1. Introduction

Canine sweat glands include apocrine and eccrine glands. Unlike apocrine glands located in the dermis, eccrine glands exist only on the footpads. Sweat gland tumors often originate from apocrine glands. Most of these tumors usually occur in the limbs, but they can develop anywhere on the body [1]. It has been reported that the incidence of apocrine gland tumors is rare in dogs (0.6–2.2% of all skin tumors), and approximately 70% of apocrine gland tumors are benign [2]. 

Malignant apocrine sweat gland tumors can occur in the head, neck, and limbs [3]. However, the most common locations of apocrine carcinomas are the axillary and inguinal areas [4]. The median postoperative survival time of dogs with apocrine adenocarcinoma is 30 months, and distant metastasis is rare in dogs [5]. A previous retrospective study reported an 8% local recurrence of apocrine sweat gland tumors in dogs [6].

Here, we present the case of a beagle dog with metastatic apocrine gland ductal adenocarcinoma. We describe the diagnosis of this case, surgical excision of the retroperitoneal metastatic apocrine gland ductal adenocarcinoma, and the short-term prognosis of the case.

## 2. Case Presentation

An 8-year-old, spayed female beagle dog, weighing 11.7 kg, was referred to the veterinary medical teaching hospital of Chungnam National University for an abdominal mass of unknown origin. The patient presented with anorexia, hypodynamia, and weight loss for a duration of 1 week. Upon physical examination, a right popliteal lymph node enlargement (20 × 30 mm, firm, and movable) and a right metatarsal skin mass (5 × 5 mm, firm, and movable) were confirmed. There were no other clinical signs related to the abdominal mass, such as abdominal distention or pain. A blood analysis did not show any specific findings, except for elevated canine C-reactive protein (10.64 mg/L; range, 0–2 mg/L).

On a radiograph, a 78 mm, round, soft tissue density mass was identified in the middle to caudal abdominal region. Abdominal ultrasonography revealed a heterogeneous mass with multifocal anechoic regions and necrotic changes. A small amount of ascites was observed around the mass. The margin of the mass was clearly distinguished from the surrounding tissues, but some parts of the mass were adjacent to the caudal pole of the right kidney. The right kidney was enlarged compared to the left kidney, and the medulla and renal pelvis were severely dilated. The proximal ureter was dilated, but the distal ureter was difficult to evaluate. The caudal vena cava was compressed by the mass, and the boundary between the vena cava and mass was unclear. 

**Figure 1:**
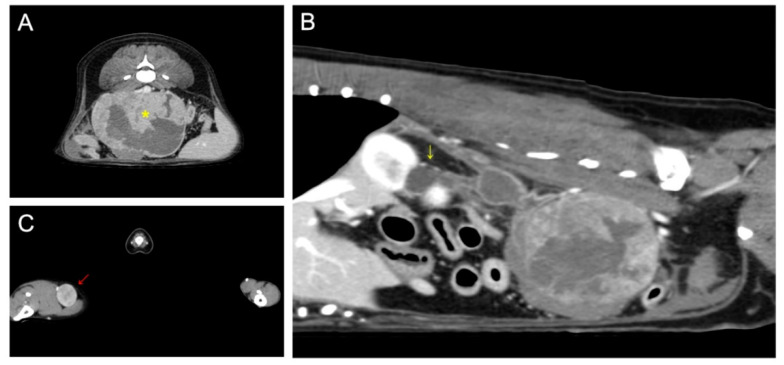
Preoperative computed tomography: (**A**) retroperitoneal mass (yellow asterisk) suspected of being metastasis from the right metatarsal skin mass; (**B**) proximal hydroureteronephrosis (yellow arrow) caused by compression of the distal ureter by the retroperitoneal tumor; (**C**) enlarged right popliteal lymph node (red arrow).

On computed tomography, a 93 × 72 × 65 mm abdominal mass with necrotic changes was closely adjacent to the caudal vena cava, abdominal aorta, right deep circumflex iliac artery, and external iliac artery (Figure 1). Severe hydroureteronephrosis was observed in the upper right urinary tract. A 22 × 19 mm right popliteal lymph node showed heterogeneous postcontrast enhancement, and the lymph node was suspected to be a metastatic lesion. Malignant glandular epithelium was confirmed on fine-needle aspiration of the right popliteal lymph node, but diagnostically valuable samples could not be obtained from the metatarsal skin mass. 

**Figure 2:**
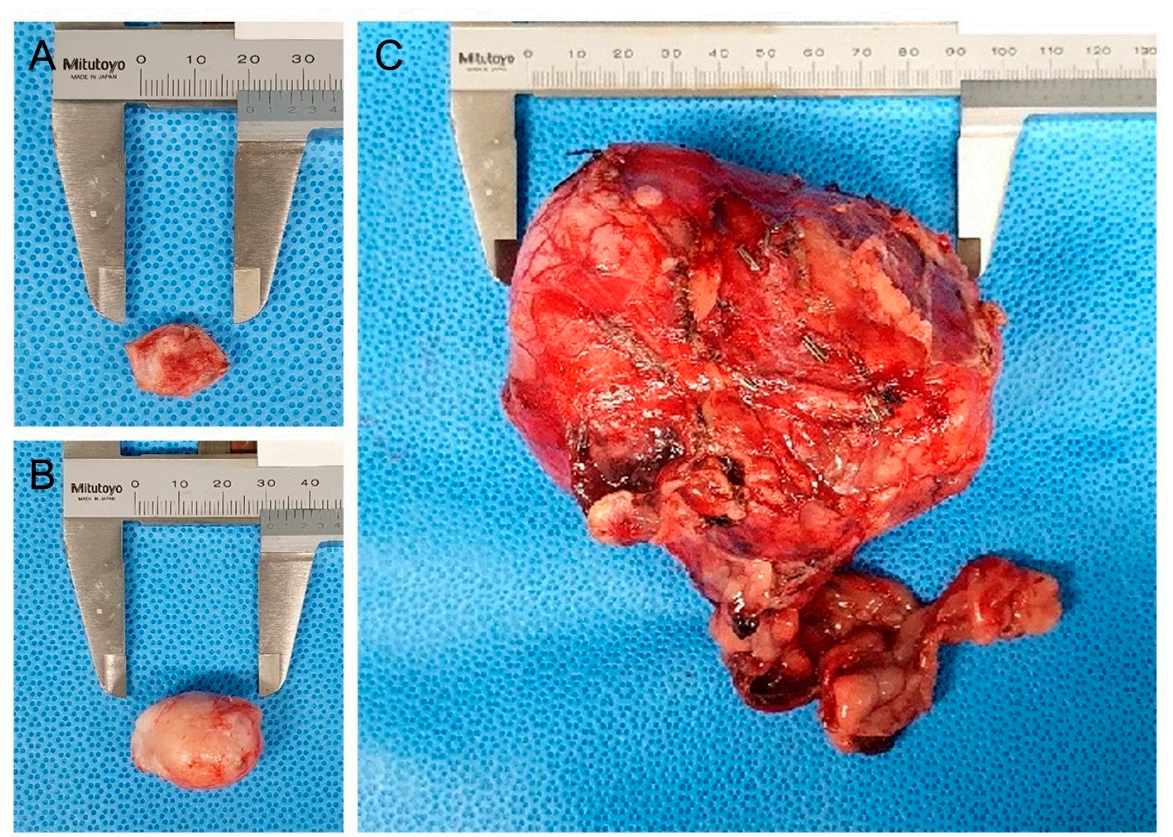
Gross appearance of the resected metatarsal skin mass, popliteal lymph node, and retroperitoneal mass: (**A**) right metatarsal region cutaneous mass resected with a 5 mm surgical margin; (**B**) resected enlarged right popliteal lymph node; (**C**) resected retroperitoneal mass.

As differential diagnoses for abdominal masses, urinary tract-derived tumors, primary retroperitoneal tumors, and metastatic tumors from the metatarsal skin mass were contemplated. Because the enlarged right popliteal lymph node and retroperitoneal mass could represent distant metastases, and considering lymphatic drainage, surgical excision of the metatarsal skin mass, popliteal lymph node, and abdominal mass was considered for histopathological examination (Figure 2). 

The dog was preoxygenated with 100% oxygen. Preanesthetic medication included maropitant (1 mg/kg, SC), cefazolin sodium (22 mg/kg, IV), diphenhydramine (2 mg/kg, SC), and dexamethasone (0.2 mg/kg, IV). Propofol (1 mg/kg, IV) and midazolam (0.2 mg/kg, IV) were slowly injected, followed by propofol (4 mg/kg, IV) for coinduction. Anesthesia was maintained with isoflurane, and constant rate infusion of remifentanil (0.1–0.3 ug/kg/min) was used for analgesia. For surgery, the patient was placed in dorsal recumbency with the right hindlimb in the hanging leg position. After the midline celiotomy, it was confirmed that the greater omentum was adhered to the mass. It was dissected to expose the mass. Some of the distal ureter was involved in the mass, and the caudal vena cava, abdominal descending aorta, and right external iliac artery were adhered to the mass. The adhered vessels were dissected from the mass using electrocautery, and the branches of the vessels were ligated with sutures or hemoclips. Because the right ureter was involved in the mass and hydroureteronephrosis was confirmed, a right ureteronephrectomy was performed. After the sufficient abdominal lavage, a vacuum suction drain was placed, and the abdominal wall was routinely closed. Right popliteal lymphadenectomy and surgical excision of the metatarsal skin mass were performed. Postoperatively, the temporary right hind limb edema was not accompanied by lameness and the patient recovered within a few days. Except for hindlimb edema, there were no postoperative complications. 

**Figure 3:**
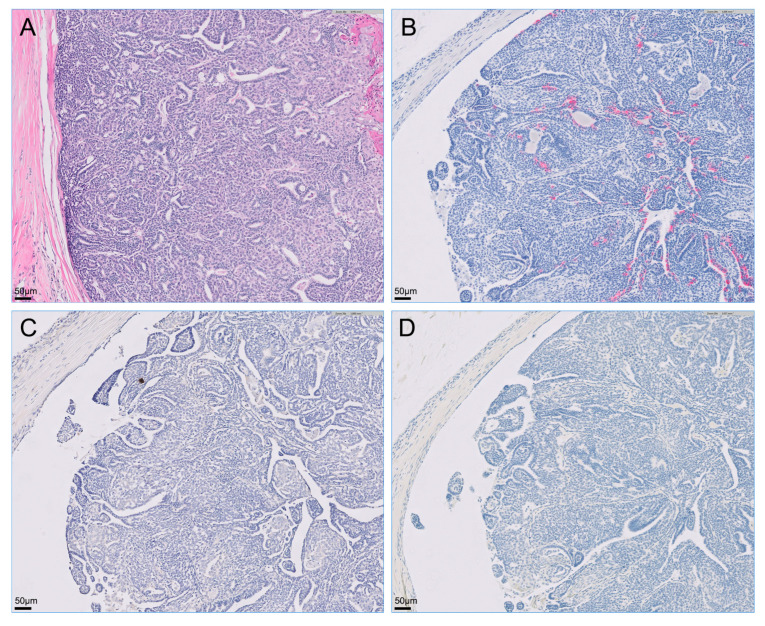
Microscopy of the right metatarsal skin mass visualized with hematoxylin-eosin staining and immunohistochemistry. Primary cutaneous tumor on the dorsum of the right metatarsal region was intensely positive for cytokeratin 7 via immunostaining but was negative for cytokeratin 20 and S100 proteins via staining on immunohistochemistry. (**A**) Hematoxylin-eosin stain; (**B**) immunohistochemistry for cytokeratin 7; (**C**) immunohistochemistry for cytokeratin 20; (**D**) immunohistochemistry for S100s.

**Figure 4:**
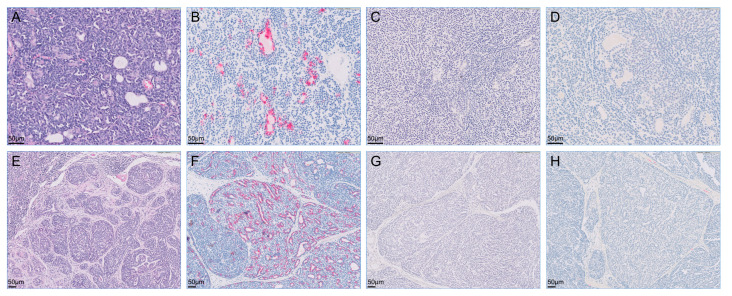
Microscopy of metastatic retroperitoneal mass (**A**–**D**) and enlarged right popliteal lymph node (**E**–**H**) visualized with hematoxylin-eosin stain and immunohistochemistry. The retroperitoneal mass and right popliteal lymph node were intensely positive for cytokeratin 7 but were negative for cytokeratin 20 and S100 proteins on immunohistochemistry. (**A**) Hematoxylin-eosin stain; (**B**) immunohistochemistry for cytokeratin 7; (**C**) immunohistochemistry for cytokeratin 20; (**D**) immunohistochemistry for S100s; (**E**) hematoxylin-eosin stain; (**F**) immunohistochemistry for cytokeratin 7; (**G**) immunohistochemistry for cytokeratin 20; (**H**) immunohistochemistry for S100s.

**Figure 5:**
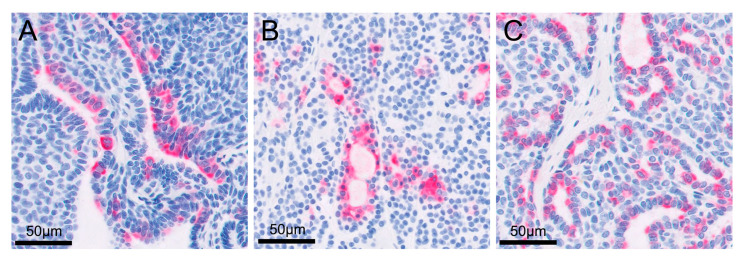
High magnification images of immunohistochemistry to show cytokeratin 7 positivity in the cytoplasm of the cells: (**A**) right metatarsal skin mass; (**B**) metastatic retroperitoneal mass; (**C**) enlarged right popliteal lymph node.

The retroperitoneal and metatarsal skin masses were histopathologically diagnosed as apocrine gland ductal adenocarcinoma. A similar cellular morphology that is most suggestive of an adenocarcinoma with highly aggressive biological behavior, indicated by evidence of metastatic spread, was observed in the retroperitoneal mass, popliteal lymph node, and metatarsal skin mass. Although evidence of lymphovascular invasion was not present in the metatarsal skin mass, the popliteal lymph node and retroperitoneal metastases showed lymphovascular invasion. Neoplastic cells were not observed in the resected right kidney. Immunohistochemically, all tumor cells were intensely positive for cytokeratin 7 but negative for cytokeratin 20 and S100 proteins (Figure 3, Figure 4 and Figure 5).

During surgery, the adhesion between the retroperitoneal tumor and the surrounding blood vessels was severe, and the surgical margin of the resected tumor was observed to be incomplete upon histopathological examination. Therefore, adjuvant chemotherapy was recommended to the patient’s owner. Unfortunately, the owners did not agree to this treatment. There were no specific problems, including local recurrence or metastasis, at the 6-month postoperative follow-up.

## 3. Discussion

Malignant apocrine gland tumors are rare in dogs. In this report, we describe the ret-roperitoneal metastasis of a primary cutaneous apocrine adenocarcinoma in a dog. The tumor was excised, and no recurrence was found in the 6-month follow-up period.

Tumors of the apocrine sweat glands usually occur in older dogs [5]. Nevertheless, the occurrence of these tumors is rare in dogs, and they account for only approximately 2% of canine skin tumors [6]. Approximately 70% of sweat gland tumors in dogs are benign in nature, but malignant tumors can recur locally and can metastasize to regional lymph nodes and the lungs [1,7]. There have been few reports of metastasis of malignant apocrine tumors in veterinary medicine. According to a previous retrospective study of 44 canine apocrine sweat gland adenocarcinomas, histopathological evidence of lymphatic infiltration was observed in 25% of cases, but only one in twenty-five dogs developed distant metastasis, and this animal was the only one with histological evidence of vascular invasion [5]. Vascular invasion was considered an important indicator of distant metastasis in the previous study. Even without vascular invasion in apocrine adenocarcinoma, however, distant metastasis seems to be possible. In the present case, although evidence of lymphovascular invasion was not present in the primary apocrine adenocarcinoma, there were the popliteal lymph node and retroperitoneal metastases.

Histopathology and immunohistochemistry are required to diagnose cutaneous apocrine adenocarcinoma [8]. At the first presentation, the retroperitoneal mass was suspected to be primary urinary tract tumors or a metastatic lesion of the medial iliac or lumbar lymph node. Histopathology revealed a partially encapsulated, multilobulated neoplasm composed of relatively monomorphic cuboidal to polygonal epithelial cells. The right popliteal lymph node was characterized as a multilobulated, partially encapsulated mass composed of epithelial cells similar to the retroperitoneal mass without remnant evidence of lymph node architecture. The retroperitoneal mass and cutaneous mass in the metatarsal region both contained a neoplasm that had similar microscopic morphology in each of the locations, suggesting that each anatomic site represented the same neoplastic process. Cytokeratins 7 and 20 are mainly distributed in the epithelia and neoplasms derived from the epithelial cells [9]. Immunohistochemical staining for cytokeratin 7 may help distinguish ductular from nonductular origins, such as primary renal tumors. It could be helpful to assess the postoperative prognosis. Median survival time was 30 months for dogs with apocrine adenocarcinoma and 16 months for dogs with renal cell carcinoma [5,10]. Most cases of adenocarcinoma are positive for cytokeratin 7 on immunohistochemical staining [9]. In addition, a previous study reported that negativity for cytokeratin 20 is important for ruling out Merkel cell carcinoma [11]. The metatarsal skin mass in this case was diagnosed as an apocrine gland ductal adenocarcinoma based on histopathology and immunohistochemistry. The metatarsal skin mass, popliteal lymph nodes, and retroperitoneal mass showed similar patchy immunoreactive patterns. Given the morphology and general similarity in immunoreactivity, the popliteal lymph node and retroperitoneal mass were considered to be metastases from the primary apocrine adenocarcinoma.

Primary cutaneous apocrine adenocarcinoma is extremely rare, even in human medicine [8]. Most of these neoplasms are indolent and slow in development, but some are rapidly progressive and sufficiently aggressive to cause distant metastasis [12]. Wide excision with regional lymphadenectomy is considered to be the only curative therapy for primary cutaneous apocrine adenocarcinoma in human and veterinary medicine [13,14]. Radiotherapy, chemotherapy, and antiandrogen therapy have been reported as therapeutic options for primary apocrine adenocarcinoma, but there is no consensus regarding the protocol due to a lack of meta-analyses to determine treatment efficacy [14,15,16]. There are some chemotherapy protocols for adenocarcinomas of other origins [17,18], but these have not yet been proven in cutaneous apocrine gland ductal adenocarcinomas. In the present case, adjuvant chemotherapy was recommended because the metastatic retroperitoneal tumor was severely adhered to adjacent blood vessels, and the surgical margin of the tumor was incomplete, but the patient’s owner refused additional treatment due to financial issues and lack of a chemotherapy protocol consensus for metastatic apocrine adenocarcinoma. 

Simko et al. and Barahak et al. reported pulmonary metastasis of cutaneous apocrine adenocarcinoma [5,13], but there is no previous case report of retroperitoneal metastasis of primary cutaneous apocrine adenocarcinoma. This case emphasizes that although primary cutaneous apocrine adenocarcinoma in dogs is rare, it carries the risk of local recurrence or distant metastasis. Therefore, wide excision is recommended for treatment, and adjuvant therapy can be considered.

## Data Availability

Data available on request due to restrictions eg privacy or ethical.
